# Herbicidal safening activity of *Loropetalum chinense* extract in alleviating pretilachlor-induced phytotoxicity in rice

**DOI:** 10.3389/fpls.2025.1711639

**Published:** 2026-01-20

**Authors:** Hongjun Chen, Xing Tang, Long Xiao, Xiu Liu, Chenzhong Jin

**Affiliations:** 1 Key Laboratory of Green Control of Crop Pests in Hunan Higher Education, Hunan Provincial Collaborative Innovation Center for Field Weeds Control, Hunan University of Humanities, Science and Technology, Loudi, China; 2 Hunan Provincial Key Laboratory of Fine Ceramics and Powder Materials, School of Materials and Environmental Engineering, Hunan University of Humanities, Science and Technology, Loudi, China

**Keywords:** alleviating phytotoxicity, herbicidal safener, *Loropetalum chinense* extract, pretilachlor, rice seedlings

## Abstract

In this study, the crude extract of natural *Loropetalum chinense* (LoE) was prepared using a simple ethanol leaching approach. Preliminary evaluations of the safening effect of LoE in both agar and soil media demonstrated its ability to protect rice seedlings from damage induced by the herbicide pretilachlor (Pre). Specifically, treatment with LoE at 50 mg/L resulted in a 40.35% increase in root length and a 25.11% increase in plant height. In contrast, treatment at 100 mg/L led to a 36.85% increase in fresh weight, demonstrating effectiveness comparable to that of the commercial safener fenclorim (Fen). Moreover, compared to rice seedlings treated with Pre alone, LoE significantly increased the levels of glutathione (GSH) and enhanced the activities of several important enzymes: glutathione S-transferase (GST), superoxide dismutase (SOD), and catalase (CAT). The mechanism of action for LoE appears to involve enhancing GST activity to accelerate the metabolism of Pre and improving herbicide tolerance by activating antioxidant enzymes. Overall, LoE shows promise as a potential eco-friendly, plant-derived herbicide safener candidate for pretilachlor. For practical commercial application, further efforts should focus on (i) clarifying the phytochemical profile of LoE extracts, and (ii) conducting in-depth assessments of LoE’s mechanisms of action, including studies on degradation kinetics and metabolite formation.

## 1 Introduction

Rice (*Oryza sativa*) and wheat (*Triticum aestivum*) are the most important cereal crops worldwide, cultivated on more than 26 million hectares in South and East Asia (Zhang et al., 2021). Studies have identified 141 weed species in paddy fields across China (Zhang, 2003; Zhang et al., 2021). Weeds pose a significant barrier to rice productivity; even with current weed management practices, average yield losses caused by weed infestations in China remain substantial 15–20% for rice and 15% for wheat (Ladha et al., 2003; Zhang, 2003; Zhang et al., 2021). Herbicides are now commonly used to deal with weeds (Zhang et al., 2021). Currently, chloroacetanilide herbicides like alachlor, acetochlor, metolachlor and pretilachlor are used in large quantities for pre-emergence control of annual grasses and broadleaf weeds in corn, soybeans, rice and many other crops (Wu et al., 2000; Sahoo et al., 2017; Rosario-Lebron et al., 2018; Woodward et al., 2018; Ding et al., 2023; Ye et al., 2023; Yu et al., 2025). However, improper herbicide use can significantly impact agricultural production, leading to crop failure and phytotoxicity in crops under field conditions (Scarponi et al., 2003; Jiang et al., 2016; Chen et al., 2021). For instance, pretilachlor applied to soil rapidly disintegrates and accumulates in crops, where it is toxic and can also harm aquatic organisms, with residues detected in crops, soil, and water (Kaur et al., 2015).

Herbicide phytotoxicity is mainly solved through several means: (i) developing selective herbicides that are relatively safe to the crops; (ii) cultivating herbicide-resistance crop plants; (iii) using herbicide safeners. Among these, applying herbicide safeners is considered the most cost-effective and widely used method. Various safeners have been developed to reduce herbicide-induced phytotoxicity in crops such as maize (*Zea mays*), grain sorghum (*Sorghum bicolor*), small-grain cereals, and rice (Deng et al., 2020c; Guo et al., 2020; Hu et al., 2020; Ye et al., 2023; Peng et al., 2024; Yu et al., 2025). These safeners typically work by either interfering with the herbicide’s interaction at its target site or enhancing the activity of detoxifying enzymes (e.g., glutathione S-transferase (GST), cytochrome P450 oxidase, superoxide dismutase (SOD) and peroxidase), thus providing selective protection to crops from herbicide damage (Persans et al., 2001; Smith et al., 2003; Hu et al., 2020; Font Farre et al., 2024). In particular, GST-catalyzed conjugation of herbicides with endogenous glutathione (GSH) transforms active xenobiotic compounds into inactive derivatives, a process that has been proven to alleviate phytotoxicity in wheat, corn, rice, and sorghum (DeRidder et al., 2002; Ye et al., 2019; Hernández Estévez and Rodríguez Hernández, 2020; Hu et al., 2020; Font Farre et al., 2024). The detoxification mechanism of chloroacetanilide herbicides is mainly linked to the levels of endogenous glutathione (GSH) and GST activity (Deng et al., 2001; Scarponi et al., 2003; Brazier-Hicks et al., 2008; Hu et al., 2020; Font Farre et al., 2024). For instance, fenclorim, a specific pyrimidine-type herbicide safener, has shown increasing rice seedlings’ tolerance to chloroacetanilide herbicides by upregulating GST expression, promoting the conjugation process with glutathione (Brazier-Hicks et al., 2008; Hu et al., 2020).

To date, approximately 20 commercialized synthetic herbicide safeners, including fenclorim, cyprosulfamide, mefenpyr-diethyl, dichlormid, flurazole, fenchlorazole, dymrone, cyometrinil, and oxabetrinil, have been marketed for crop protection (Abu-Qare and Duncan, 2002; Guo et al., 2019; Deng et al., 2020c; Deng et al., 2020a; Zhao et al., 2023; Pingarron-Cardenas et al., 2024; Sun et al., 2024). However, some commercially available herbicide safeners (e.g., dichlormid, benoxacor, and furilazole) are toxic to both aquatic organisms and mammals (Sivey et al., 2015; Deng et al., 2020c; Zhao et al., 2023). Additionally, the synthesis of these chemical safeners often involves harmful organic solvents and complicated processes, highlighting the urgent need for the development of more efficient, cost-effective, and environmentally friendly alternatives.

Natural substances, particularly certain plant extracts, represent a promising avenue for discovering new herbicide safeners. Z-ligustilide and senkyunolide A, isolated from *Ligusticum chuanxiong*, as well as the sanshool mixture extracted from *Sichuan pepper* fruits, have shown impressive herbicide-safening effects against metolachlor in rice (Li et al., 2013; Tang et al., 2014; Li et al., 2016). These findings provide a reference for the development of plant-derived herbicide safeners. Flavonoids, which are known for their antioxidant properties, also play a role in interacting with specific genes involved in detoxification (Bais et al., 2003; Kim and Jang, 2009; De Martino et al., 2012; Zhang et al., 2023). The isoflavonoids formononetin and biochanin A have been shown to exert herbicide-safening effects on sorghum against the herbicide imazaquin (Siqueira et al., 1991). *Loropetalum chinense*, a traditional Chinese medicinal plant distributed in the subtropical regions of East Asia, exhibits hemostatic and antidiarrheal properties. It mainly contains essential oils, tannins, flavonoids, and lignins (Liu et al., 1997; Zhang et al., 2013; Chen et al., 2018). The flavonoids in *L. chinense* extracts include kaempferol, quercetin, myricetin, myricetin-3-O-α-L-rhamnoside, and isoquercitrin (Fu et al., 2012), and the total flavonoid content extracted from its tender leaves, old leaves, and flowers has been reported to range from 3.82% to 7.44% (Qiu et al., 2015). Thus, it is reasonable to infer that the crude extract of *L. chinense* may have the potential to be used as a plant-derived herbicide safener for crops.

This study explored the effectiveness of *L. chinense* crude extract (LoE) in safeguarding rice seedlings from phytotoxicity caused by the herbicide pretilachlor. The research assessed the impact of pretilachlor on key physiological metrics of seedlings and compared the safening activity of LoE to that of fenclorim. It also delved into how LoE influenced detoxifying compounds and enzyme activities related to its protective effects. The findings indicated that LoE demonstrated promising safening activity, positioning it as a suitable candidate for further exploration as a natural herbicide safener for pretilachlor in rice cultivation.

## 2 Materials and methods

### 2.1 Material and chemicals

The paddy rice seeds (Tianyou 3301 purchased from Hunan Hybrid Rice Research Center) chosen as the experimental crop were cultivated in a SPX-250-GB incubator (Hangzhou, China). The pretilachlor (purity=95%, Pre) was obtained from Shandong Qiaochang Chemical Co., Ltd. (Shandong, China). Commercially available bioactive ingredient detection kit including glutathione (GSH), glutathione S-transferase (GST), superoxide dismutase (SOD), Catalase (CAT) and peroxidase (POD) were purchased from Nanjing Jiancheng Bioengineering Institute (Nanjing, China). Tween-80 were purchased from Jiangsu Haian Petroleum Chemical Plant (Jiangsu, China). Ethanol, agar and fenclorim were purchased from Aladdin Biochemical Technology Co., Ltd. (Shanghai, China). Methanol and acetone (chromatographic grade) was all purchased from Tianjin Kemiou Chemical Reagent Co., Ltd. (Tianjin, China). All reagents were of analytical reagent grade and used without further purification. Ultrapure water obtained from a Eped-Plus-E3 system (18.2 MΩ·cm) was used in the study. The young stem and leaves of *L.chinense* were collected from Luxi Town, Jishou City, Hunan Province.

### 2.2 Preparation of *L.chinense* crude extract

The preparation of LoE was conducted as follows: First, the young stems and leaves of *L. chinense* were dried, thoroughly ground, and passed through a 40-mesh sieve to collect the powder. Next, 300 g of the collected powder was immersed in 4.5 L of ethanol solution (ethanol/water = 9:1) for 24 hours and then filtered. The filtrate was retained for subsequent steps, while the filter residue was subjected to ultrasonic extraction with 3 L of 90% ethanol for 15 minutes, followed by filtration to collect the filtrate. Finally, all filtrates were combined and concentrated by evaporation to obtain a pasty LoE (9.0 g) with an extraction efficiency of 3%. The extract was stored at 4 °C for further use. These extraction steps were repeated as necessary, combining the resulting LoE products after each batch, until a sufficient quantity of extract was accumulated to complete all planned experimental procedures.

### 2.3 Phytotoxicity of pretilachlor or LoE toward rice cultured in agar medium

The phytotoxicity of pretilachlor (Pre) on the growth of rice seedlings was investigated using an agar medium. Prior to rice cultivation, rice seeds were first disinfected, soaked in clean water for 24 hours, then placed in Petri dishes lined with double-layer filter paper, and germinated under appropriate conditions. The agar medium for rice cultivation was prepared as follows: Agar was added to a beaker with water, heated to form a 0.3% agar aqueous solution, and cooled to approximately 50 °C. Subsequently, 5 mL of pretilachlor or LoE stock solution was mixed with 95 mL of the above agar solution in a beaker, stirred thoroughly, and cooled to room temperature to obtain a series of experimental agar media supplemented with Pre or LoE for rice cultivation. The final experimental concentrations of pretilachlor were 0.01, 0.04, 0.08, 0.16, 0.32, and 0.64 mg/L, while the concentrations of LoE were set at 25, 50, 100, 200, and 400 mg/L. In particular, a 0.1% Tween 80 aqueous solution treatment was set as the control.

Subsequently, rice seeds that had germinated to the whitening stage were transplanted into 500 mL beakers containing the pretilachlor or LoE supplemented agar medium, with 10 seeds per beaker. The beakers with rice seeds were incubated in an incubator under a 13 h:11 h (dark:light, light intensity 10000 lux) illumination cycle at a temperature of 28 ± 1 °C and a relative humidity of 60%. After 7 days of cultivation, 6 seedlings were randomly selected from each beaker (excluding rotten seeds) to record data on root length, bud length, and fresh weight of the plants. It should be noted that all experiments were performed in triplicate to ensure better reproducibility, and the bioassay results presented are the averages of the three replicates. The phytotoxicity of Pre or LoE on the growth indices of rice seedlings was evaluated, and the inhibition rates of Pre on root length, plant height, and fresh weight were calculated using Equation 1.

(1) $$inhibition\;\;rate(\%)=\frac{control-treated\;with\;Pre\;or\;LoE}{control}\times 100$$

### 2.4 Evaluation of safening activities of LoE in agar medium

The herbicide safening activity of LoE was evaluated under laboratory conditions following the method described in the literature (Li et al., 2013). Uniformly germinated rice seedlings were selected and transplanted into 0.3% agar media for the primary screening test, with the media containing the following treatments: 0.32 mg/L Pre alone, and 0.32 mg/L Pre combined with different concentrations of LoE (25, 50, 100, 200, and 400 mg/L), respectively. The agar medium treated with a 0.1% Tween 80 aqueous solution served as the control, while the combination of 0.32 mg/L Pre and 0.08 mg/L fenclorim (Fen) was used for comparison. The beakers containing rice seedlings were incubated in an incubator under a 13 h:11 h (dark:light, light intensity 10000 lux) photoperiod, at a temperature of 28 ± 1 °C and a relative humidity of 60%. After 7 days of cultivation, 6 seedlings were randomly selected from each beaker (excluding rotten seeds) to record data on root length, bud length, and fresh weight, which are indicators related to herbicide safening activity. It should be noted that all experiments were performed in triplicate to ensure good reproducibility, and the bioassay results presented are the averages of the three replicates. The relative injury recovery rates induced by LoE, which reflect the herbicide safening effects on root length, plant height, and fresh weight, were calculated using Equation 2.

(2) $$injury\;re\mathrm{cov}ery\;rate(\%)=\frac{treated\;with\;safener+Pre-treated\;with\;Pre}{control-treated\;with\;Pre}\times 100$$

### 2.5 Effect of pretilachlor on the physiological indexes of rice seedlings

First, a series of pretilachlor (Pre) stock solutions with concentrations of 9.96, 4.48, 2.24, 1.12, 0.56, 0.28, and 0.14 mg/L were prepared. Then, 100 mL of each prepared Pre stock solution was mixed with 200 g of soil to obtain a series of pretilachlor-amended soils with final concentrations of 4.48, 2.24, 1.12, 0.56, 0.28, 0.14, and 0.07 mg/kg, respectively. A 0.1% Tween-80 aqueous solution was used as the control. Subsequently, rice seeds that had germinated to the whitening stage were sown in pots containing the Pre-amended soil, with 10 seeds per pot. The pots were then placed in an incubator under a 13 h:11 h (dark:light, light intensity 10000 lux) photoperiod at a temperature of 28 ± 1 °C and a relative humidity of 60%. After 7 days of cultivation, 6 seedlings were randomly selected from each pot (excluding rotten seeds) to record data on plant length and fresh weight. The phytotoxicity of Pre on the growth indices of rice seedlings grown in soil was evaluated, and the inhibition rates of Pre on plant height and fresh weight were calculated using Equation 1.

### 2.6 Evaluation of safening activities of LoE by pot method

The safening activity of LoE on the growth of soil-cultivated rice seedlings was evaluated using the aforementioned method. Uniformly germinated rice seedlings were selected and transplanted into soil media for the experiment, with the media subjected to the following treatments: 0.56 mg/kg Pre alone, and 0.56 mg/kg Pre combined with different dosages of LoE (50, 100, 200, 400, and 800 mg/kg). Soil treated with a 0.1% Tween 80 aqueous solution served as the control, while soil treated with 0.56 mg/kg Pre combined with 0.3 mg/kg Fen was used for comparison. The pots were then incubated in an incubator under a 13 h:11 h (dark:light, light intensity 10000 lux) photoperiod at a temperature of 28 ± 1 °C and a relative humidity of 60%. After 7 days of cultivation, 6 seedlings were randomly selected from each pot (excluding rotten seeds) to record data on plant length and fresh weight, which are indicators related to herbicide safening activity. The relative injury recovery rate induced by LoE, which reflects the herbicide safening effect, was calculated using Equation 2.

### 2.7 Assays of GSH content and related enzymes activities

After 7 days of treatment in agar medium, rice seedling samples were collected from the following groups: the pretilachlor (Pre) alone group (0.32 mg/L), the LoE + Pre group (50 mg/L LoE combined with 0.32 mg/L Pre), the Fen + Pre group (0.08 mg/L fenclorim (Fen) combined with 0.32 mg/L Pre), and the control group (treated with 0.1% Tween 80 aqueous solution) for comparison. Prior to enzyme activity assessment, 0.4 g of rice leaves were ground into powder in liquid nitrogen, suspended in extraction buffer, and centrifuged at 4000 rpm for 20 minutes to obtain the homogenized supernatant.

The glutathione (GSH) content and glutathione S-transferase (GST) activity were determined following the instructions of commercial kits (Nanjing Jiancheng Bioengineering Institute, Nanjing, China). The GSH content was measured by recording the absorbance at 420 nm and calculated by comparison with a standard of known concentration, using 2,2’-dithiosalicylic acid as the colorimetric indicator. It was expressed as milligrams per gram of protein (mg/g protein). GST activity was measured by recording the absorbance at 412 nm based on the amount of CDNB/GSH conjugate catalyzed by GST, and expressed as units per milligram of protein (U/mg protein). Total superoxide dismutase (SOD) activity was determined using the xanthine/xanthine oxidase method, which is based on the production of O_2_^-^ anions that induce color changes in the reagent at 550 nm. SOD activity was expressed as units per milligram of protein (U/mg protein). Catalase (CAT) activity was evaluated by measuring the decomposition of H_2_O_2_ at a UV absorption wavelength of 405 nm, and expressed as units per gram of protein (U/g protein). Peroxidase (POD) activity was determined based on the change in absorbance at 420 nm due to the catalysis of H_2_O_2_, with the enzyme activity expressed as units per milligram of protein (U/mg protein).

### 2.8 Evaluation of phytotoxicity and safening activities of quercetin

The phytotoxicity of quercetin on the growth of rice seedlings cultured in agar medium was investigated. Briefly, 0.3% agar media treated with different concentrations of quercetin (6.25, 12.5, 50, and 100 mg/L) were used to cultivate rice seedlings for 7 days, and growth parameters including root length, plant height, and fresh weight of the seedlings were recorded. Agar medium treated with a 0.1% Tween 80 aqueous solution served as the control. The herbicide safening activity of quercetin was evaluated following the aforementioned method. Specifically, uniformly germinated rice seedlings were selected and transplanted into 0.3% agar media for the experiments, with the media containing either 0.32 mg/L Pre alone or 0.32 mg/L Pre combined with different concentrations of quercetin (6.25, 12.5, 50, and 100 mg/L). Notably, treatment with a 0.1% Tween 80 aqueous solution was set as the control, while treatment with 0.32 mg/L Pre combined with 0.08 mg/L Fen was used for comparison. After 7 days of cultivation, growth parameters of the rice seedlings (including root length, plant height, and fresh weight) were collected, and the herbicide safening activity of quercetin was evaluated based on these parameters.

### 2.9 Statistical analyses

All data were statistically analyzed using SPSS software (version 26.0) and expressed as mean ± standard error of at least three replicate data points for each measurement method. Three different batches of rice plants were repeatedly tested for the purpose of determining growth indexes. Prior to analysis, the assumptions for ANOVA, including normality and homogeneity of variances, were assessed. Significant differences between treatment methods were identified using Duncan’s test at a significance level of p < 0.05.

## 3 Results and discussion

### 3.1 Safening effects of LoE on the growth of rice seedlings in agar medium

**Figure 1:**
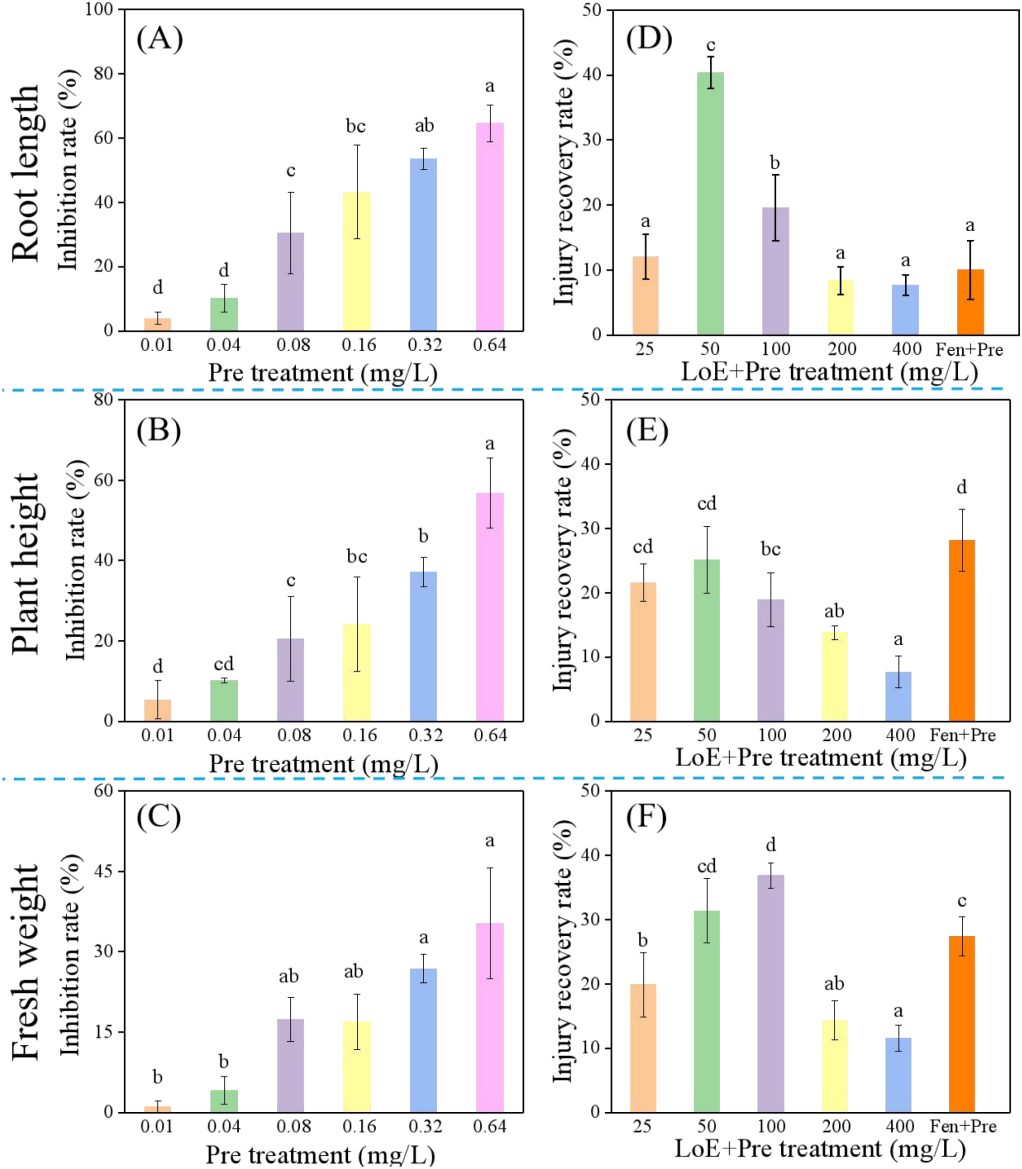
Phytotoxicity of different concentrations of pretilachlor on the root length **(A)**, plant height **(B)** and fresh weight **(C)** of rice seedlings after treated for 7 days. Herbicide safening activities of different concentrations of LoE on root length **(D)**, plant height **(E)** and fresh weight **(F)** of rice seedlings after 7 days co-treatment with Pre (0.32 mg/L). For comparison, the commercially available herbicide safener Fen (0.08 mg/L) combined with Pre (0.32 mg/L) was included as a treatment. All rice seedlings were grown in agar medium under greenhouse conditions.

Pretilachlor (Pre) has been reported to cause phytotoxicity, reducing rice fresh weight by 7% when applied at 1350 g ha^–1^ in paddy filed (Shen et al., 2013). The concentration of herbicides is crucial for evaluating the herbicide safening efficacy of compounds (Wu et al., 2000; Chen et al., 2021). Therefore, the phytotoxic effects of Pre on the growth of rice seedlings cultured in agar medium were investigated, and the results are presented in **Figure 1**. As shown in **Figures 1A-C**, Pre significantly inhibited the growth indicators of rice seedlings, including root length, plant height, and fresh weight, indicating that these growth parameters can serve as theoretical evaluation indices for safening activity. When the herbicide concentration ranged from 0.01 to 0.64 mg/L, the inhibition rate on rice seedlings increased with the elevation of Pre concentration. In particular, at a Pre concentration of 0.64 mg/L, the inhibition rates reached 64.59% for root length, 56.78% for plant height, and 35.31% for fresh weight, respectively. Notably, when the Pre concentration exceeded 0.32 mg/L, rice seedlings exhibited obvious chlorosis and yellowing of leaves, indicating severe inhibition of their growth. Based on the data of various growth indicators of rice seedlings, the concentration of Pre used in the subsequent assessment of LoE’s safening activity was selected as 0.32 mg/L.

The herbicide safening activity of LoE was evaluated, and the results are shown in **Figures 1D-F**. LoE exhibited positive recovery rates for the growth indicators of rice seedlings (root length, plant height, and fresh weight) across all experimental concentration ranges, demonstrating that it alleviated Pre-induced damage to rice plants and confirming its potential to protect rice seedlings from Pre phytotoxicity. Specifically, the recovery rates of all indicators first gradually increased to the highest level and then decreased. The maximum recovery rates were 40.35% for root length and 25.11% for plant height at the LoE concentration of 50 mg/L, and 36.85% for fresh weight at 100 mg/L LoE. In addition, the commercial safener fenclorim (Fen) showed recovery rates of 10.01% for root length, 28.13% for plant height, and 27.43% for fresh weight, respectively. Compared with Fen, the maximum recovery rate of LoE was nearly 4-fold higher for root length and 1.3-fold higher for fresh weight, suggesting that LoE has excellent safening activity against Pre. To ensure adequate safening effects, the concentration of LoE in practical applications in agar medium should be controlled within the range of 50–100 mg/L.

The phytotoxicity of LoE on the growth of rice seedlings in agar medium was examined, and the results are shown in **Supplementary Figure S1**. Surprisingly, within the experimental concentration range, LoE only slightly inhibited the root length, plant height, and root number of rice seedlings while increasing their fresh weight, indicating that LoE treatment did not significantly suppress the growth of rice seedlings. Given its superior safening activity against Pre and low phytotoxicity to rice seedlings, it can be inferred that LoE has the potential to serve as a safener.

### 3.2 Enzyme activity assessment

**Figure 2:**
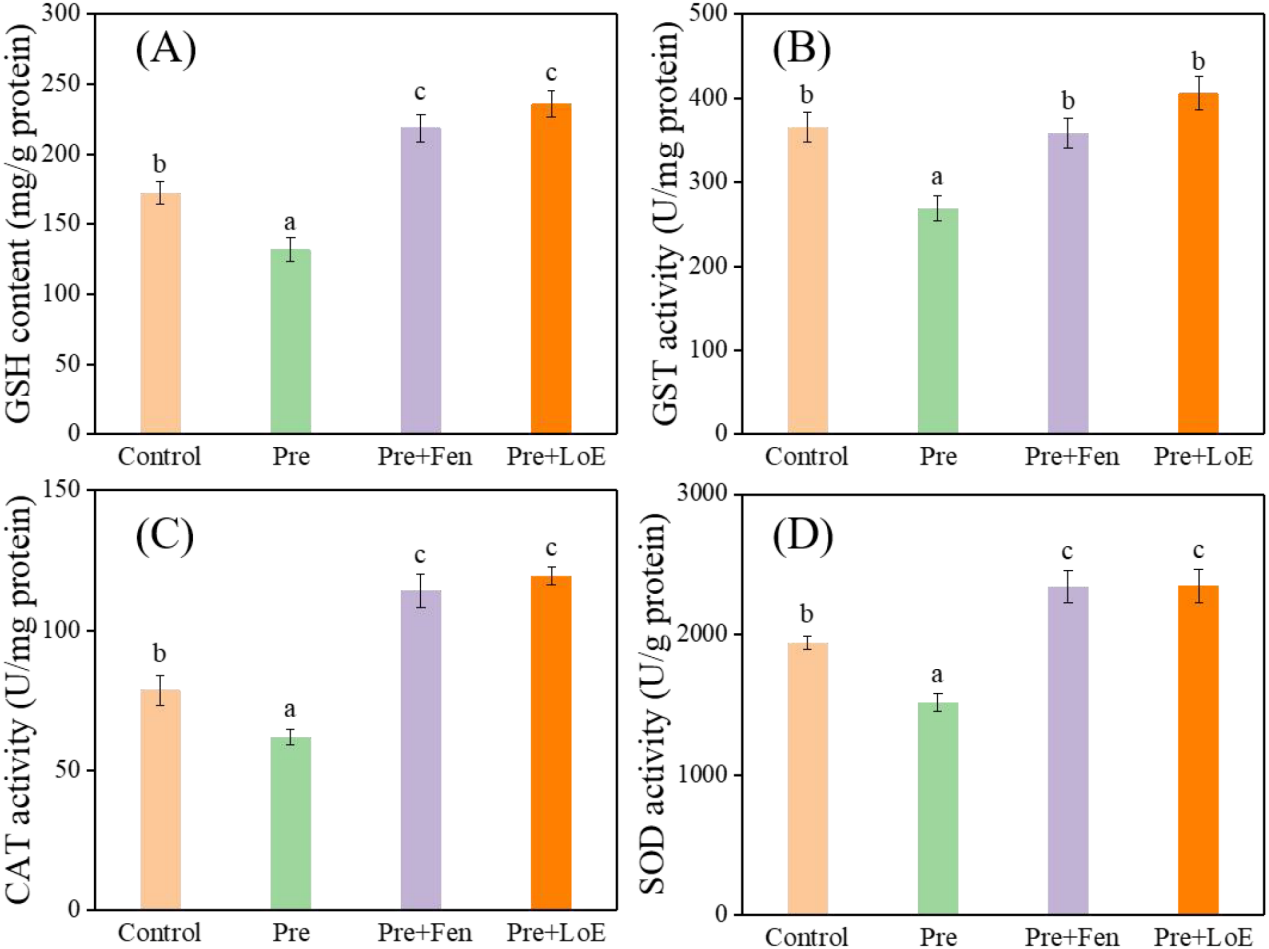
Protective effects on GSH content **(A)**, GST activity **(B)**, CAT activity **(C)** and SOD activity **(D)** in rice seedlings treated with Pre (0.32 mg/L), LoE (50 mg/L) combined with Pre (0.32 mg/L), and Fen (0.08 mg/L) combined with Pre (0.32 mg/L). Note: Rice seedlings were cultivated in agar medium under greenhouse conditions, with 0.1% Tween-80 aqueous solution treatment serving as the control.

It is well established that GST in crop plants such as rice, corn, and wheat can be induced by safeners to varying degrees, thereby increasing crop tolerance to herbicides. Several commercial safeners (benoxacor, fenclorim, and furilazole) and natural product safeners (Z-ligustilide, isopimpinellin, and melatonin) have been reported to enhance GST activity. For example, treatment with the safener fenclorim has been shown to increase GST activity in rice plants to 1.3–1.9 times that of the control (treated only with pretilachlor) (Scarponi et al., 2003; Sivey et al., 2015; Deng et al., 2020a; Deng et al., 2020b). Therefore, the levels of GSH content and GST activity in rice seedlings treated with LoE and Pre were analyzed. As shown in **Figures 2A, B**, Pre treatment reduced both GSH content and GST activity in rice seedlings compared to the control. In contrast, Fen significantly increased the GSH content by 65.9% and GST activity by 33.3% relative to Pre alone, confirming that Fen’s detoxification mechanism involves enhancing herbicide metabolism in crops, as previously reported in the literature (Deng et al., 1995; Hu et al., 2020). Similarly, LoE treatment resulted in significant increases in GSH content and GST activity—78.8% and 50.9% higher, respectively, than in seedlings treated with Pre alone—comparable to the effects observed with Fen. These findings suggest that LoE may protect rice seedlings from pretilachlor toxicity by enhancing the metabolic detoxification of the herbicide in rice.

Plants have evolved mechanisms to minimize herbicide toxicity through their antioxidant systems. Antioxidant enzyme activities, such as those of superoxide dismutase (SOD), peroxidase (POD), and catalase (CAT), have been reported to be associated with herbicide tolerance in various plant species (Deng et al., 2020b). As shown in **Figures 2C, D**, and **Supplementary Figure S2**, compared with the control, the activities of CAT, SOD, and POD (**Supplementary Figure S2**) in rice seedlings treated with Pre alone decreased by approximately 21.3%, 21.9%, and 7.3%, respectively. Additionally, compared with seedlings treated with Pre alone, the addition of LoE increased CAT and SOD activities in rice seedlings by 93.0% and 55.1%, respectively, while reducing POD activity by 11.0%. These results indicate that LoE may enhance the herbicide tolerance of rice seedlings to Pre by increasing antioxidant enzyme activities.

### 3.3 Evaluation of herbicide safening activities of quercetin

**Figure 3:**
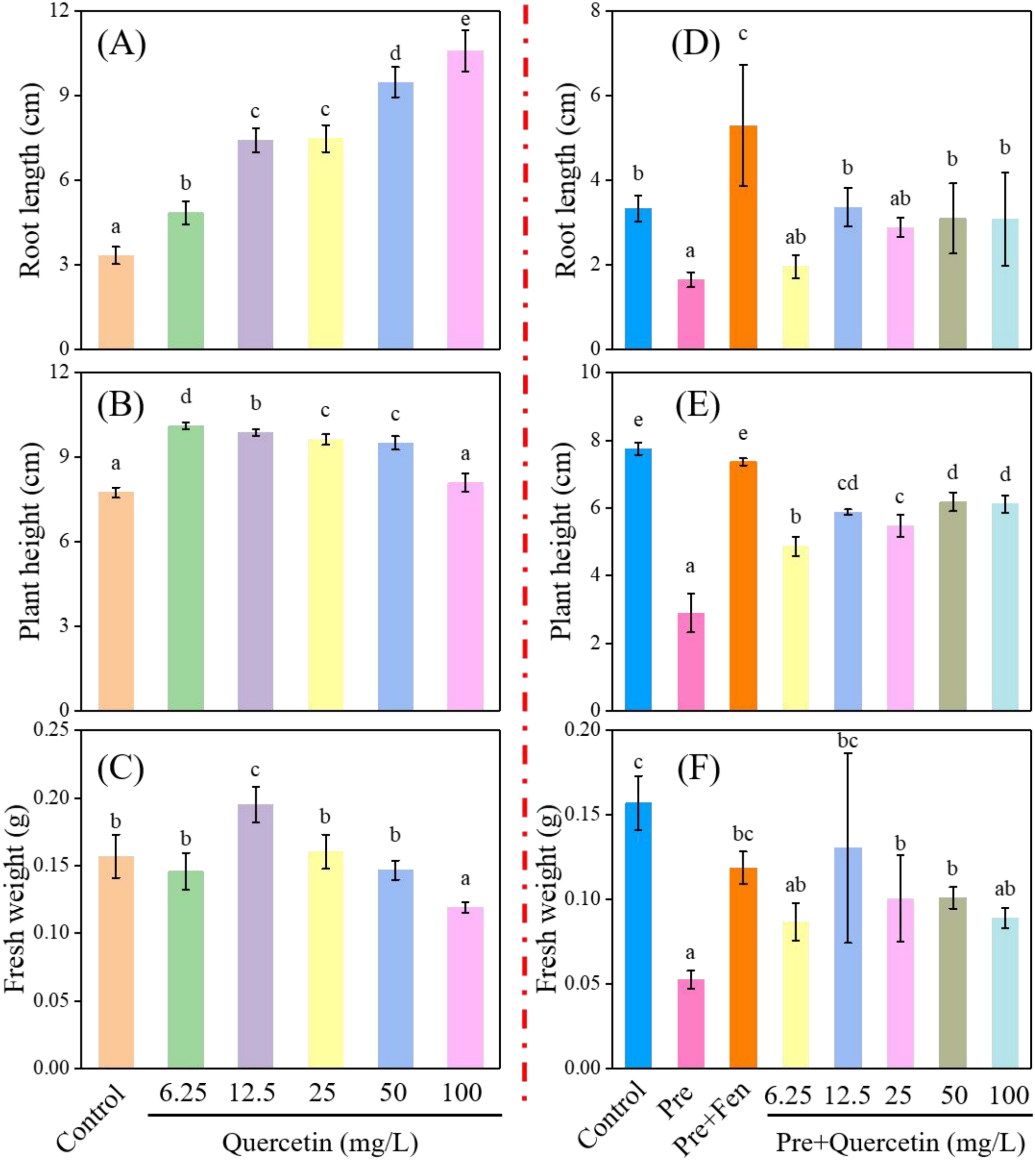
Effects of different concentrations of quercetin on root length **(A)**, plant height **(B)**, and fresh weight **(C)** of rice seedlings after 7 days of treatment. Safening activities of different concentrations of quercetin on root length **(D)**, plant height **(E)**, and fresh weight **(F)** of rice seedlings were assayed after 7 days of co-treatment with Pre (0.32 mg/L). For comparison, the treatment with Fen (0.08 mg/L) combined with Pre (0.32 mg/L) was included, and the 0.1% Tween-80 aqueous solution treatment served as the control. All rice seedlings were cultured in agar medium under greenhouse conditions.

It has been reported that the total flavonoid content in ethanol extracts of *Loropetalum chinense* varies significantly, with flowers containing 7.44%, young leaves 4.75%, and old leaves 3.82% (Qiu et al., 2015). In addition, quercetin—a type of flavonoid—was successfully isolated and identified. The average quercetin content in the leaves ranged from 0.146% to 0.168%, while the flowers contained 0.962% (Zhang et al., 2013; Chen et al., 2018). Therefore, the phytotoxicity and safening activity of quercetin against Pre were investigated, with the results shown in **Figure 3**. Compared with the control, quercetin increased the root length of rice seedlings in a concentration-dependent manner (**Figure 3A**). When its concentration was lower than 12.5 mg/L, it increased the plant height and fresh weight of rice seedlings, whereas high concentrations exerted inhibitory effects (**Figures 3B, C**). According to previous reports (Bais et al., 2003; De Martino et al., 2012). the inhibitory effects on plant height and fresh weight were likely attributed to the dose-related phytotoxicity of quercetin. Meanwhile, quercetin could promote the growth of rice seedlings at low treatment concentrations, indicating its potential safening activity against Pre.

Subsequently, the safening activity of quercetin on the growth of rice seedlings was evaluated. As shown in **Figures 3D-F**, compared with rice seedlings treated with Pre alone, treatment with 12.5 mg/L quercetin increased the root length, plant height, and fresh weight of rice seedlings by 102.0%, 61.5%, and 80%, respectively. These results indicate that quercetin has a detoxifying effect on Pre. In other words, the safening activity of quercetin may be one of the factors contributing to the detoxifying effect of LoE. It is also evident that the safening activity of quercetin was inferior to that of Fen, which increased the root length, plant height, and fresh weight of rice seedlings by 216.8%, 92.1%, and 68%, respectively. It should be emphasized that although quercetin has been proven to protect rice seedlings from Pre damage, the specific compounds responsible for the safening activity of LoE still need to be further identified. It is recommended to evaluate the detoxifying capacity of other flavonoids and phenolic compounds present in the crude extract to determine the association and contribution of these active ingredients.

### 3.4 Identification protective effects of LoE on rice seedling by pot method

Nowadays, a variety of natural safeners extracted from Chinese medicinal herbs have been discovered, such as sanshools, echinacea alkylamides, benzofurans, Z-ligustilide, senkyunolide, bergapten and isopimpinellin (Deng, 2022a; Deng, 2022b). Unfortunately, none have been commercialized or launched into the market due to several limitations: (i) they have lower activity compared to commercial safeners and therefore need to be used in larger doses (Zeng et al., 2019); and (ii) some natural safeners are difficult to synthesize and have high extraction costs, including CO_2_ supercritical fluid extraction (Li et al., 2016) or supercritical chromatographic extraction (Tang et al., 2014).

**Figure 4:**
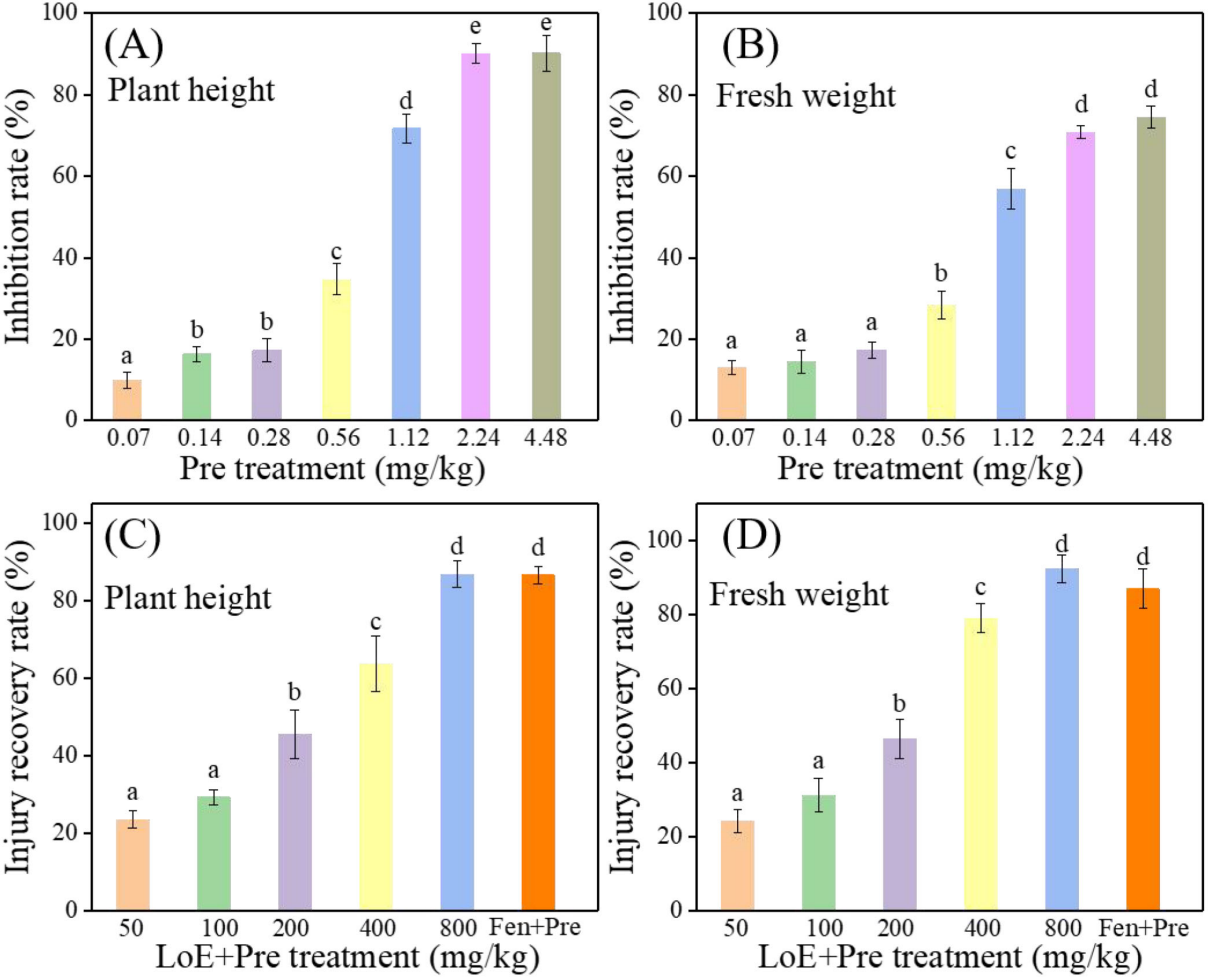
Phytotoxicity of different concentrations of pretilachlor on plant height **(A)** and fresh weight **(B)** of rice seedlings after 7 days of treatment. Safening effect of different concentrations of LoE on plant height **(C)** and fresh weight **(D)** of rice seedlings after 7 days of co-treatment with LoE and Pre (0.56 mg/kg). The treatment with Fen (0.3 mg/kg) combined with Pre (0.56 mg/kg) was used as a positive control. Notes: All rice seedlings were cultivated in soil.

For practical applications, the safening effect of LoE on rice seedlings under soil cultivation conditions were investigated. As shown in **Figures 4A, B**, Pre exerted concentration-dependent inhibitory effects on the growth of rice seedlings, with the inhibition rate increasing as the Pre concentration rose. In particular, when the Pre concentration was 0.56 mg/kg, it significantly inhibited the growth of rice seedlings compared with the control, with inhibition rates of 34.74% for plant height and 28.33% for fresh weight, respectively. Additionally, when the Pre concentration exceeded 1.12 mg/kg, toxic symptoms appeared on the leaves of rice plants. Therefore, the concentration of Pre was set to 0.56 mg/kg in the subsequent pot experiments to test the safening activity of LoE. As depicted in **Figures 4C, D**, LoE treatment with Pre (0.56 mg/kg) resulted in a concentration-dependent recovery rate in rice seedlings. At an LoE concentration of 800 mg/kg, the recovery rates for rice seedling growth reached the highest, at 86.92% for plant height and 92.51% for fresh weight. In comparison, the recovery rates for plant height and fresh weight under Fen (0.3 mg/kg) treatment were 86.7% and 87.1%, respectively. Notably, there was no significant difference between the highest recovery rates induced by LoE and Fen, indicating that LoE exhibits comparable safening activity against Pre in soil-cultivated rice.

Although the required usage of LoE (800 mg/kg) is higher than that of Fen (0.3 mg/kg), LoE demonstrates a concentration-dependent recovery rate and comparable safening performance, highlighting its potential for commercial application. Importantly, the preparation process for plant-sourced LoE is straightforward, requiring only harmless ethanol as a solvent. Using LoE as a safener instead of Fen is therefore more eco-friendly, contributing to reduced organic pollution and aligning with the goals of green, safe, and environmentally sustainable agricultural production. For practical commercial application, further efforts should focus on (i) clarifying the phytochemical profile of LoE extracts, and (ii) conducting in-depth assessments of LoE’s mechanisms of action, including studies on degradation kinetics and metabolite formation.

## 4 Conclusions

In summary, LoE was prepared using a simple ethanol extraction method. Preliminary tests in both agar and soil-based rice cultivation demonstrated that LoE could protect rice seedlings from the phytotoxicity of the herbicide Pre. Notably, LoE exhibited herbicide safening activity by increasing GSH content and enhancing the activities of GST, SOD, and CAT enzymes in rice seedlings. The protective mechanism of LoE appears to involve not only accelerating the metabolism of Pre through enhanced GST activity but also improving rice herbicide tolerance by boosting antioxidant enzyme activity. Additionally, quercetin identified as a functional compound in LoE, was shown to protect rice seedlings from Pre-induced damage. However, the specific compounds responsible for the overall safening activity of LoE still need to be fully identified. It is recommended to evaluate the detoxifying capacity of other flavonoids and phenolic compounds present in the crude extract to determine their association and contributions. Overall, LoE shows promise as a potential eco-friendly, plant-derived herbicide safener candidate for pretilachlor. For practical commercial application, further efforts should focus on (i) clarifying the phytochemical profile of LoE extracts, and (ii) conducting in-depth assessments of LoE’s mechanisms of action, including studies on degradation kinetics and metabolite formation.

## Data Availability

The original contributions presented in the study are included in the article/**Supplementary Material**. Further inquiries can be directed to the corresponding author.
