# Supplementary material for: Herbicidal safening activity of Loropetalum chinense extract in alleviating pretilachlor-induced phytotoxicity in rice
Source: Front Plant Sci. 2026 Jan 20;16:1711639. doi: 10.3389/fpls.2025.1711639 (PMC12864381; doi:10.3389/fpls.2025.1711639)
Supplement: Supplementary file 1 [file SupplementaryFile1.docx]

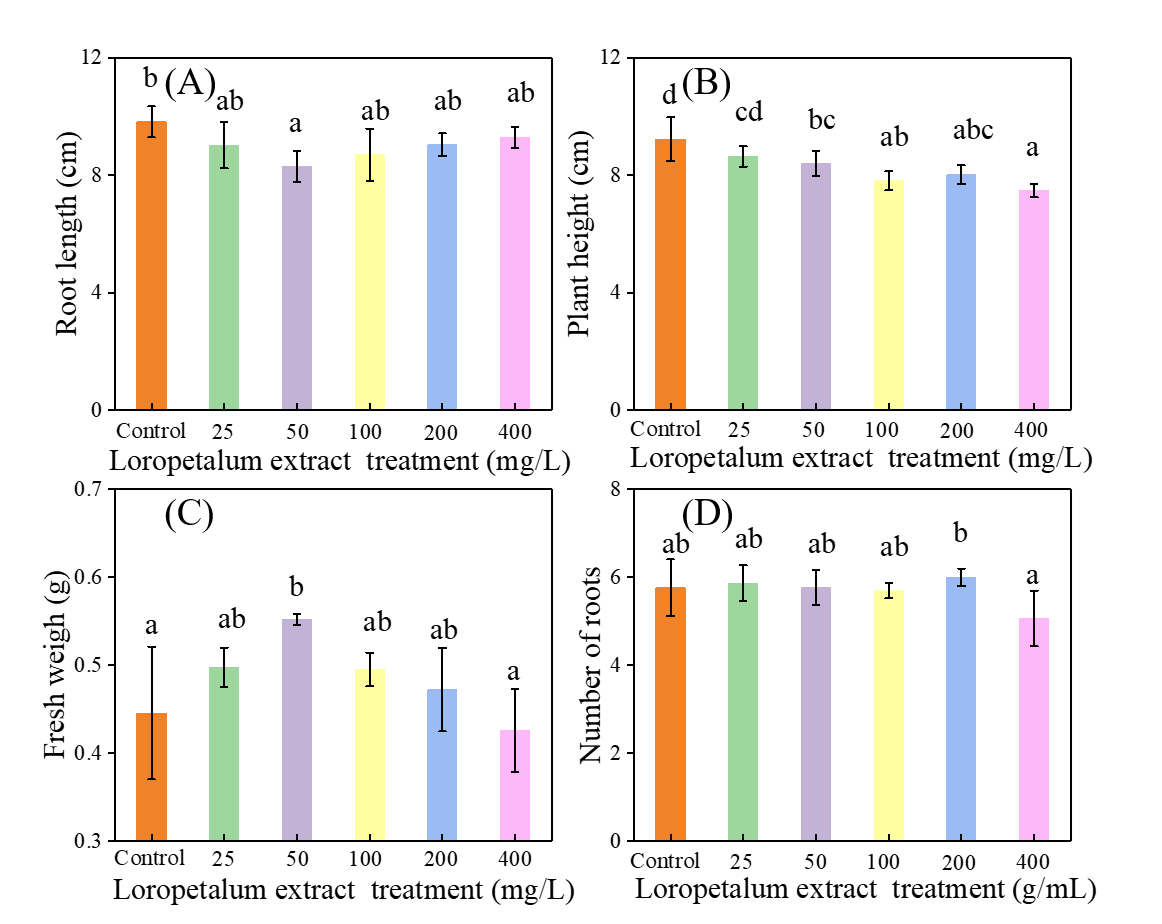


Figure S1. Phytotoxicity of different concentrations of loropetalum extract on the root length (A), bud length (B), plant fresh weight (C) and number of roots (D) of rice seedlings after treated for 7 days using agar culture methodology, where 0.1% Tween 80 aqueous solution treatment were set as the control. Data are presented as the mean ± standard error of triplicate experiments. Different letters in each figure show significant differences at the p< 0.05 level through Duncan’s multiple range test using SPSS Statistics 26.0.


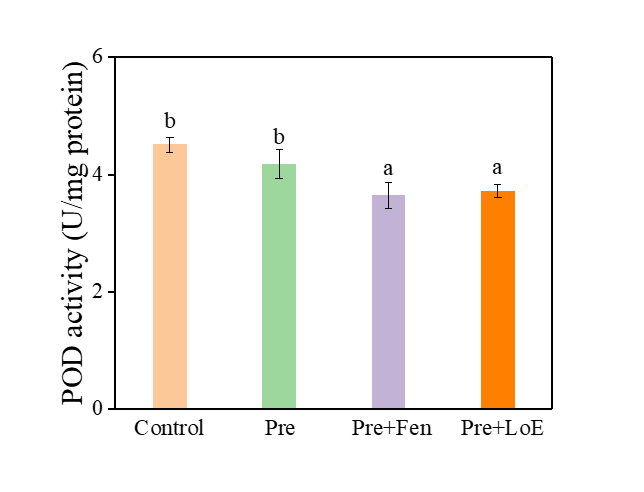


Figure S2. Protective effects on the antioxidant enzyme POD activity of rice seedlings treated with Pre (0.32 mg/L), LoE (50 mg/L) combined with Pre (0.32 mg/L), and Fen (0.08 mg/mL) combined with Pre (0.32 mg/L). Note: the rice seedlings were cultivated under greenhouse conditions in agar medium, and 0.1% Tween-80 aqueous solution treatment was used as the control. Data are presented as the mean ± standard error of triplicate experiments. Different letters in each figure show significant differences at the p< 0.05 level through Duncan’s multiple range test using SPSS Statistics 26.0.
